# Military Sanitation

**Published:** 1906-06-16

**Authors:** 


### The Hospital

A Journal of

The Medical Sciences and Hospital Administration.

Vol. XL.—No. 1,029. SATURDAY, JUNE 16 1906.

### MILITARY SANITATION.

The action of Mr. Brodrick and Sir Frederick Treves, in directing attention through the *Times* to the chronic and continuing inefficiency of our military medical system as a means of protecting armies in the field against disease, and to the contrast which it offers to that which has been brought into operation by our allies the Japanese, is of a kind which ought to be adequately supported alike in Parliament and by external public opinion. Mr. Brodrick rightly claims that, by reforms introduced under his administration, the Medical Department of the Army has been rescued from the unpopularity in the profession by which it was previously weighed down, and that it now attracts candidates of a high class, in numbers considerably exceeding the vacancies to be provided for. The expenditure on the Medical Vote, which in 1898 was £295,000, is now £490,000; the medical officers have risen from 832 to 1,002; and the Medical College will shortly be in full work for the instruction of officers. The "humanitarian problem of tending the sick and wounded has been faced; but the regulations of the British service as to disease prevention remain substantially what they were 100 years ago." Two diseases alone, dysentery and enteric fever, caused 74,000 admissions to hospital and 9,200 deaths in South Africa; and both these diseases are in medical opinion largely preventible by a system which would provide for the isolation of those affected, by a scientific system of excreta disposal, and a satisfactory water supply. The difficulty of obtaining these things, according to Mr. Brodrick, depends upon the fact that the army surgeon, although a health officer, has no sanitary staff. He is responsible, but he can give no orders. He can only act through a commanding officer, often junior to him, who has no technical knowledge. The remedy suggested is that the combatant officers should be taught, and taught by the medical officers, "the elements of military hygiene"; and that every cadet at Sandhurst or Woolwich should be examined on passing out "in a problem which he could grasp as easily as tactics or strategy." In other words Mr. Brodrick proposes to replace the existing ignorance of the combatant officers by the self-conceit of smatterers, and to afflict the unfortunate army surgeon with colleagues or superiors who, instead of merely ignoring his recommendations, as they do at present, would fancy themselves qualified to criticise them.

Sir Frederick Treves, as might be expected from his professional knowledge, while he agrees with Mr. Brodrick as to the vital importance of the problem, takes quite a different view of the manner in which alone it could be solved. He sees clearly that the first thing needful is to place the Medical Department in a position to enforce its recommendations, and that the next is to give sufficient instruction to the fighting elements to enable them intelligently to second the efforts made for their preservation. He quotes with approval Surgeon-Major Seaman's statement that "Japan organised her medical department on broad generous lines, and gave its representatives the rank and power their great responsibilities merit, recognising that they had to deal with a foe that kills 80 per cent. of the total mortality. She even had the temerity (strange as it may seem to an English or American Army official) to grade her medical men as high as the officers of the line who combat the enemy that kills only 20 per cent." Sir Frederick goes on to mention the reforms which seem to him to be necessary; and his description of them affords an amusing contrast to Mr. Brodrick's proposal to inflict sanitary instruction on the already hard-worked cadets at Woolwich and Sandhurst. He declares that the head of the Army Medical Department should be a medical man, and not, as at present, the Adjutant-General; and that the Director-General should be responsible for the efficiency and economical administration of the department in all its branches, with control of the money voted for the medical service. Next, and manifestly most important of all, that the Army medical officer should be vested with such authority and provided with such *personnel* as will enable him to carry out those sanitary arrangements in the field which experience has proved to be absolutely essential to secure the minimum loss of life from disease. Last of all, he asks that the combatant officer should have as a part of his qualifications some knowledge of hygiene as applied to campaigning and barrack life; and that a like knowledge, of a more elementary character, should be possessed by the private soldier.

It is impossible to read these communications, and still more impossible to read them between the lines, without perceiving that the difficulties to which they point have their origin in the same condition to which Dr. Johnson once imputed a mistake of his own—that is to say, "pure ignorance." The class of people who furnish members of Parliament, and officers of State, and "combatant" officers of the Army, have grown up, generally speaking, in an atmosphere from which all effective knowledge of physical science has been excluded, and in which questions regarded as debatable have been usually settled by some form of compromise. They would probably admit that the 74,000 admissions to hospital and the 9,200 deaths from prenventible disease in South Africa were to be regretted; and they would plead, with helpless imbecility, that the recommendations of the Medical Department had been carried out to some extent, and as far as was compatible with the traditions of the service, or with the views of a regimental commanding officer who regarded sanitary precautions as "sheer nonsense." It is here, indeed, that we have the crux of the whole question. It is not sufficient that there should be, somewhere in a subordinate branch of the Army, the knowledge by which epidemics of disease can be prevented; but this knowledge, in order to render it effective, must be placed in a position of authority, such that its depositaries are able to enforce the observance of the precautions which they recommend. This, and nothing less than this, is what has been done by Japan; it has been done effectively in campaigns of previously unexampled magnitude, and has been not only compatible with, but largely contributory to, the attainment of military successes to which no parallels can be found in history.

